# Mammary Tumorigenesis and Metabolome in Male Adipose Specific Monocyte Chemotactic Protein-1 Deficient MMTV-PyMT Mice Fed a High-Fat Diet

**DOI:** 10.3389/fonc.2021.667843

**Published:** 2021-09-09

**Authors:** Lin Yan, Sneha Sundaram, Bret M. Rust, Matthew J. Picklo, Michael R. Bukowski

**Affiliations:** U.S. Department of Agriculture, Agricultural Research Service, Grand Forks Human Nutrition Research Center, Grand Forks, ND, United States

**Keywords:** adipose MCP-1, MMTV-PyMT, plasma metabolome, diet, male, mice

## Abstract

Male breast cancer, while uncommon, is a highly malignant disease. Monocyte chemotactic protein-1 (MCP-1) is an adipokine; its concentration in adipose tissue is elevated in obesity. This study tested the hypothesis that adipose-derived MCP-1 contributes to male breast cancer. In a 2x2 design, male MMTV-PyMT mice with or without adipose-specific *Mcp-1* knockout [designated as *Mcp-1^-/-^* or wild-type (WT)] were fed the AIN93G standard diet or a high-fat diet (HFD) for 25 weeks. *Mcp-1^-/-^* mice had lower adipose *Mcp-1* expression than WT mice. Adipose *Mcp-1* deficiency reduced plasma concentrations of MCP-1 in mice fed the HFD compared to their WT counterparts. *Mcp-1^-/-^* mice had a longer tumor latency (25.2 weeks *vs.* 18.0 weeks) and lower tumor incidence (19% *vs.* 56%), tumor progression (2317% *vs.* 4792%), and tumor weight (0.23 g *vs.* 0.64 g) than WT mice. Plasma metabolomics analysis identified 56 metabolites that differed among the four dietary groups, including 22 differed between *Mcp-1^-/-^* and WT mice. Pathway and network analyses along with discriminant analysis showed that pathways of amino acid and carbohydrate metabolisms are the most disturbed in MMTV-PyMT mice. In conclusion, adipose-derived MCP-1 contributes to mammary tumorigenesis in male MMTV-PyMT. The potential involvement of adipose-derived MCP-1 in metabolomics warrants further investigation on its role in causal relationships between cancer metabolism and mammary tumorigenesis in this male MMTV-PyMT model.

## Introduction

Breast cancer in men accounts for roughly 1% of all breast cancer cases (1). However, breast cancer is an aggressive disease in men. Approximately, 90% of all breast cancer diagnosed in men are invasive carcinoma (2), and 25% of male breast cancer patients have distant metastasis at the time of clinical presentation (3). Additionally, results from the Surveillance, Epidemiology, and End Results (SEER) program show that improvements in breast cancer survival for men lag behind that for women (4).

Similar to female breast cancer, male breast cancer is classified into various subtypes including luminal b (5), an aggressive subtype of a higher grade with poorer prognosis (6) that accounts for 21% of breast cancer in men (5). The mouse mammary tumor virus-polyoma middle T antigen (MMTV-PyMT) model is a commonly used model in research of luminal b breast cancer (7). The MMTV-PyMT model conserves many of the defining characteristics of human subtypes (7). Compared to females, male MMTV-PyMT mice exhibit a delayed onset of palpable mammary tumors with a lower penetrance of metastasis (8). This delayed onset mimics clinical observations that breast cancer in men occurs approximately five to 10 years later than the average age of breast cancer occurrence in women (9).

Obesity is a major risk factor for breast cancer. Obese breast cancer patients often present high-grade lesions, elevated risk of recurrence, and increased incidence of lymph node involvement and metastasis (10, 11). Body fat accumulation, a hallmark of obesity (12, 13), may account for this association. Adipose tissue produces proinflammatory adipokines, including monocyte chemotactic protein-1 (MCP-1), that are elevated by obesity and contribute to obesity-related diseases.

MCP-1 is a major member of the adipokine family (14). In response to obesity, adipocytes increase the production of MCP-1 leading to obesity-induced inflammation (14–16). Clinical studies show that an elevation in MCP-1 occurs with cancer progression and has prognostic value for breast cancer. Poor outcomes and short disease-free intervals are related to high levels of MCP-1 in breast cancer patients (10, 17, 18). Silencing the expression of *Mcp-1* or its receptor protects mice against obesity-mediated inflammation in visceral adipose tissue (19, 20) and inhibits mammary tumor growth and metastasis in MDA-MB-231 mice (21). Depletion of MCP-1 reduces mammary tumorigenesis in C3(1)/SV40Tag mice (22) and spontaneous metastasis of Lewis lung carcinoma in C57BL/6 mice (23).

We have reported that adipose specific *Mcp-1* knockout reduces high-fat diet-enhanced mammary tumorigenesis in female mice (24) and metastasis of Lewis lung carcinoma (25) in male mice. However, the role of adipose-derived MCP-1 in male breast cancer remains unelucidated. We hypothesized that adipose-derived MCP-1 contributes to mammary tumorigenesis in male mice. The present study tested this hypothesis by investigating the effects of adipose specific MCP-1 deficiency on mammary tumorigenesis in male MMTV-PyMT mice fed a high-fat diet.

## Materials and Methods

### Animals and Diets

The Grand Forks Human Nutrition Research Center vivarium provided mice for this study. The breeders were obtained from The Jackson Laboratory (Bar Harbor, ME, USA). Hemizygous male MMTV-PyMT mice on an FVB background were bred to female C57BL/6 mice with both alleles of the MCP-1 exons 2-3 flanked by loxP sites (MCP-1^fl/fl^). Adipose tissue-specific knockout of MCP-1 was achieved by breeding male mice homozygous for MCP-1^fl/fl^ and heterozygous for the PyMT oncogene (MMTV-PyMT^+^/MCP-1^fl/fl^) with female MCP-1^fl/fl^ mice expressing Cre recombinase driven by the adiponectin promoter (MCP-1^fl/fl^/Adipoq-Cre^+^). Male mice heterozygous for the PyMT oncogene carrying two floxed MCP-1 alleles and positive for Cre expression (MMTV-PyMT^+^/MCP-1^fl/fl^/Adipoq-Cre^+^) were designated as adipose *Mcp-1* knockout (*Mcp-1^-/-^*) mice. Male littermates that were negative for Cre expression (MMTV-PyMT^+^/MCP-1^fl/fl^/Adipoq-Cre^-^) served as wild-type (WT) controls. All mice used in this study were on a combination of the FVB and C57BL/6 backgrounds. Mice were maintained in a pathogen-free room on a 12:12-hour light/dark cycle with a temperature of 22 ± 1°C. The standard AIN93G diet (26) and a high-fat diet (HFD) providing 16% and 45% of energy from soybean oil, respectively, were used in this study (**Table 1**). Both diets were powder diets and were stored at −20°C until feeding. The study was performed in accordance with the Guide for the Care and Use of Laboratory Animals by the National Institutes of Health (27).

**Table 1:** Composition of diets.

Ingredient	AIN93G	High-fat
	g/kg	g/kg
Corn Starch	397.5	42.5
Casein	200	239
Dextrin	132	239
Sucrose	100	120
Soybean oil	70	239
Cellulose	50	60
AIN93 mineral mix	35	42
AIN93 vitamin mix	10	12
L-Cystine	3	3.6
Choline bitartrate	2.5	3
*t*-Butylhydroquinone	0.014	0.017
Total	1000	1000
Energy	%	%
Protein	20	20
Fat	16	45
Carbohydrate	64	35
Analyzed gross energy kcal/g^a^	4.3 ± 0.1	5.2 ± 0.1

a Values are means ± SEM of three samples analyzed from each diet using oxygen bomb calorimeter (Model 6200; Oxygen Bomb Calorimeter, Parr Instrument, Moline, IL, USA).

### Experimental Design

Mice were weaned onto the AIN93G diet at three weeks of age. Following one week of acclimation with the AIN93G diet, WT and *Mcp-1^-/-^* mice each were randomly assigned into one of four groups (32 per group for WT and 34 per group for *Mcp-1^-/-^* mice fed the AIN93G and HFD, respectively). Mice were housed two per cage to avoid stress related to single housing and weighed weekly. Food intake (12 mice per group) was recorded daily for three consecutive weeks one week after the initiation of the HFD. Body composition of conscious, immobilized mice was assessed one week prior to the end of the study by using the Echo Whole Body Composition Analyzer (Model 100, Echo Medical Systems, Houston, TX, USA). The percent body fat mass was calculated by using the formula: (fat mass/body mass) x 100; the percent body lean mass was obtained by using the formula: (lean mass/body mass) x 100.

### Measurement of Mammary Tumors

Mice were palpated for mammary tumors twice weekly. Tumor latency was defined as the age at which the first mammary tumor was detected (28). Palpable tumors were measured weekly by using a digital caliper (Fred V Fowler Company, Newton, MA, USA). Tumor volume was calculated by using the formula: length x width^2^ x 0.5 (28). Tumor progression was defined as the percentage change in tumor volume over time and calculated by using the formula: [(end volume – start volume)/start volume] x 100 (29). End volume was the tumor volume measured at the end of the study; start volume was the volume of the palpable tumor when it was first detected.

### Tissue Harvest

At termination, mice were fasted for six hours before they were euthanized with an intraperitoneal injection of a mixture of ketamine and xylazine followed by exsanguination. Mammary tumors were collected and weighed. Epididymal adipose tissue and plasma were harvested and stored at -80°C. Lungs were collected and fixed in Bouin’s solution for assessing the extent of metastasis (30).

### RNA Isolation and Real-Time Quantitative PCR

Total RNA was isolated from epididymal adipose tissue by using the RNeasy Lipid Tissue Mini Kit following the manufacturer’s protocol (Qiagen, Germantown, MD, USA). The quality and quantity of RNA were analyzed by using Nanodrop 8000 Spectrophotometer (Thermo Scientific, Wilmington, DE, USA). cDNA was synthesized by using the High Capacity cDNA Reverse Transcription Kit (Applied Biosystems, Waltham, MA, USA) following the manufacturer’s protocol. Real-time qPCR of *Mcp-1* was analyzed and normalized to the 18s rRNA by using the TaqMan Assay of Demand primers on the ABI QuantStudio 12K-Flex Real-time PCR system (Applied Biosystems). The 2^-ΔΔCT^ method was used to calculate the relative changes in gene expression (31).

### Quantification of MCP-1 in Plasma

Sandwich enzyme-linked immunosorbent assay (ELISA) kit was used to quantify MCP-1 (R&D Systems, Minneapolis, MN, USA) in plasma following the manufacturer’s protocol. Samples were read within the linear range of the assay. The accuracy of the analysis was confirmed by using the controls provided in the kit.

### Metabolomics Analyses

Metabolomics analysis was conducted on plasma samples from mice without palpable mammary tumors (n = 10 per group) (32, 33). This was because few mice developed palpable tumors, particularly *Mcp-1^-/-^* mice. Samples were extracted and derivatized by silylation methyloximation and analyzed by gas chromatography time-of-flight mass spectrometry (GC-TOF-MS) for untargeted metabolomics of primary metabolism. The analysis was performed and obtained data were processed by using the BinBase database (34) at the West Coast Metabolomics Center (University of California-Davis, Davis, CA, USA). Unidentified peaks were removed from the dataset and excluded from the subsequent analysis. For the remaining identified compounds, quantifier ion peak heights were normalized to the sum intensities of all known compounds. Compounds representing less than 0.02% of total signal intensity for identified compounds were excluded from statistical analysis. Additional compounds were excluded if they could not be identified as either an intermediate species or metabolic endpoint common to mammalian metabolism based upon the Kyoto Encyclopedia of Genes and Genomes (KEGG) Database or the Human Metabolome Database (35–37).

### Statistical Analyses

Two-way analysis of variance (ANOVA) and Tukey contrasts were performed to examine the effects of diet (AIN93G or HFD), genotype (WT or *Mcp-1^-/-^*), and their interactions on *Mcp-1* expression in adipose tissue, MCP-1 concentration in plasma, body weight, body composition, and energy intake among the four dietary groups. The LIFEREG procedure was used to fit the Lognormal model to latency data. The LIFETEST procedure was used to produce the Kaplan-Meier plots. Results are reported as means ± standard error of the mean (SEM); tumor latency is reported as medians and 95% confidence intervals (95% CI). Data were analyzed by using SAS 9.4 (SAS Institute, Cary, NC, USA). Differences with a *p* ≤ 0.05 are considered significant.

Metabolomics analyses were performed by using MetaboAnalyst 5.0 (McGill University, Sainte Anne de Bellevue, Quebec, Canada). Data were scaled by Pareto scaling method and analyzed by sparse partial least square-discriminant analysis (sPLSDA) (38, 39). Hierarchical clustering heatmap was constructed by using the normalized peak intensity with Euclidean distance for distance measurement and the Ward error sum of squares hierarchical clustering methods for Cluster algorithm. Group averages were reported for the top 25 metabolites identified. The MACRO procedure (SAS 9.4) was used to examine effects of diet, genotype, and their interactions on changes in plasma metabolites with the false discovery rate-corrected *p*-values reported. Results of metabolomics analyses from treatment groups are presented as fold changes in comparison to the WT control group fed the AIN93G diet.

Pathway and network analyses were performed by using MetaboAnalyst 5.0 (40). Pathway analysis of alterations in metabolic pathways in MMTV-PyMT mice was performed by using the pathway library for *Mus musculus* according to the KEGG database (41). Pathway enrichment analysis coupled with pathway topology analysis was performed to identify the altered metabolic pathways. Obtained *p* values from the pathway enrichment analysis were adjusted by the Holm method (42). Network analysis was performed to map the functional relationships of identified metabolites between the AIN93G and HFD, between WT and *Mcp-1^-/-^* mice, and between WT and *Mcp-1^-/-^* mice fed the HFD by using the KEGG Global Metabolic Network and the Metabolite-Metabolite Interaction Network. Differences with a *p* ≤ 0.05 are considered significant.

## Results

### Body Weight, Adipose *Mcp-1* Expression, and Plasma MCP-1 Concentrations

**Figure 1:**
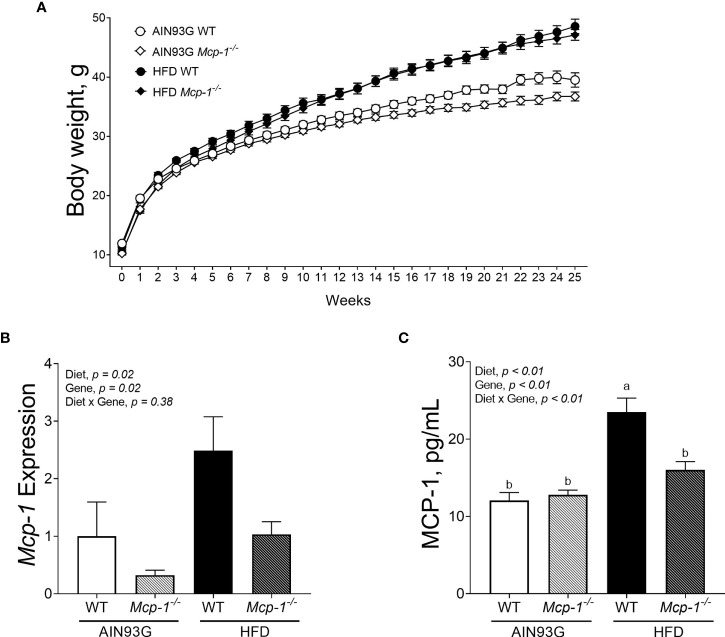
Body weight **(A)**, adipose *Mcp-1* expression **(B)**, and plasma concentrations of MCP-1 **(C)** in MMTV-PyMT mice with or without adipose monocyte chemotactic protein-1 knockout [*Mcp-1^-/-^* or wild-type (WT)] and fed the AIN93G or high-fat diet (HFD). Mice fed the HFD were heavier than mice fed the AIN93G diet; the difference was significant three weeks after the HFD (*p* < 0.05). *Mcp-1^-/-^* mice fed the AIN93G diet were smaller than their WT counterparts; the difference was significant for the last four weeks of the study (*p* < 0.05). Values are means ± SEM [n = 32 per group for WT mice, n = 34 per group for *Mcp-1^-/-^* mice for **(A)**; n = 10 per group for **(B)**; n = 16 per group for **(C)**]. Values with different letters are significant at *p* ≤ 0.05 for **(C)**.

Mice fed the HFD were heavier than mice fed the AIN93G diet, regardless of genotype (**Figure 1A**). The difference was significant after three weeks on the HFD and remained for the remainder of the study (*p* < 0.05) (**Figure 1A**). *Mcp-1^-/-^* mice fed the AIN93G diet were smaller than their WT counterparts; the difference was significant in the last four weeks of the study (*p* < 0.05) (**Figure 1A**).

The HFD elevated *Mcp-1* expression in adipose tissue by 166% compared to the AIN93G diet, regardless of genotype (**Figure 1B**). Adipose *Mcp-1* knockout diminished *Mcp-1* elevation by 61% compared to WT mice, regardless of diet (**Figure 1B**). Plasma concentrations of MCP-1 from WT mice fed the HFD were 95% higher than that from WT mice fed the AIN93G diet (**Figure 1C**). Adipose *Mcp-1* deficiency prevented plasma MCP-1 elevation in HFD-fed *Mcp-1* ^-/-^ mice, which did not differ from that of AIN93G-fed *Mcp-1^-/-^* mice (**Figure 1C**).

### Body Composition and Energy Intake

**Figure 2:**
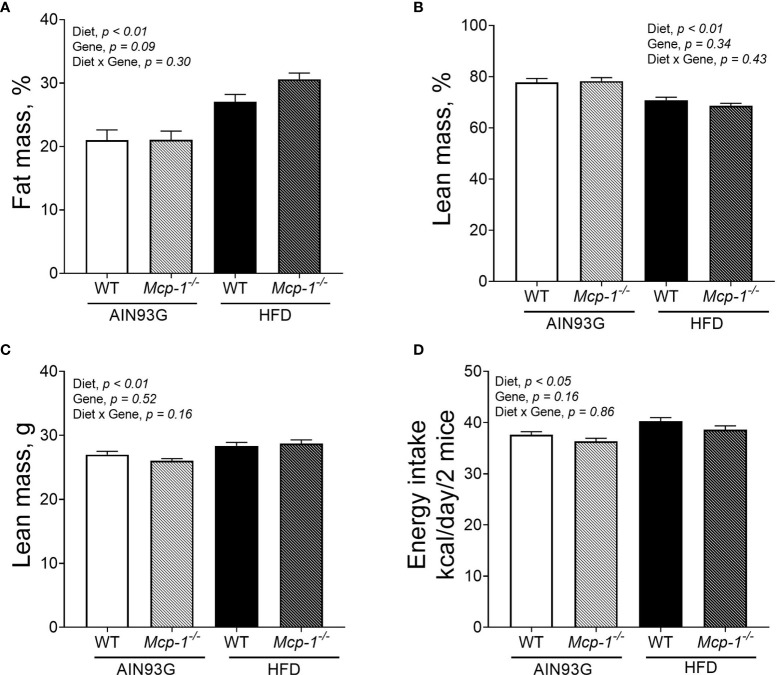
The percent body fat mass **(A)**, percent body lean mass **(B)**, absolute lean mass **(C)**, and energy intake **(D)** of MMTV-PyMT mice with or without adipose monocyte chemotactic protein-1 knockout [*Mcp-1^-/-^* or wild-type (WT)] and fed the AIN93G or high-fat diet (HFD). Values are means ± SEM [n = 32 per group for WT mice, n = 34 per group for *Mcp-1^-/-^* mice for **(A–C)**; n = 12 mice per group for **(D)**].

Regardless of genotype, the percent body fat mass of mice fed the HFD was 37% greater than that of mice fed the AIN93G diet (28.8 ± 0.9% *vs.* 21.1 ± 0.9%) (**Figure 2A**). The percent body lean mass of the HFD-fed mice was 11% less than that of the AIN93G-fed mice (69.7 ± 0.9% *vs.* 77.9 ± 0.9%) (**Figure 2B**). The absolute lean mass of mice fed the HFD was slightly higher than that of mice fed the AIN93G diet (28.5 ± 0.4 g *vs.* 26.6 ± 0.4 g) (**Figure 2C**). Energy intake of the HFD-fed mice was 7% higher than that of the AIN93G-fed mice (39.4 ± 0.7 kcal per day *vs.* 37.0 ± 0.7 kcal per day) (**Figure 2D**). There were no significant differences in percent body fat mass, percent body lean mass, absolute lean mass, and energy intake between WT and *Mcp-1^-/-^* mice, regardless of diet (**Figures 2A–D**).

### Mammary Tumorigenesis and Lung Metastasis

Fewer *Mcp-1^-/-^* mice developed palpable mammary tumors than WT mice. The tumor incidence was 19% for *Mcp-1^-/-^* mice (13 out of 68 mice) and 56% for WT mice (36 out of 64 mice) (*p* < 0.01), regardless of diet. There was no difference in tumor incidence between the HFD (26 out of 66 mice) and the AIN93G diet (23 out of 66 mice), regardless of genotype.

**Figure 3:**
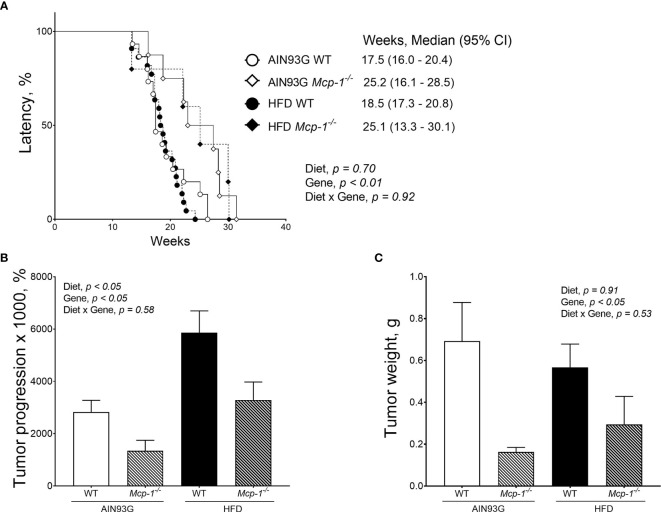
Mammary tumor latency **(A)**, tumor progression **(B)**, and tumor weight **(C)** of MMTV-PyMT mice with or without adipose monocyte chemotactic protein-1 knockout [*Mcp-1^-/-^* or wild-type (WT)] and fed the AIN93G or high-fat diet (HFD). Values are means ± SEM [(n = 5-22 per group) for **(B, C)**]. N = 32-34 per group for **(A)**.

Palpable mammary tumors were detected later in *Mcp-1^-/-^* mice than in WT mice. Tumor latency of *Mcp-1^-/-^* mice fed the AIN93G and HFD was 25.2 weeks and 25.1 weeks, respectively (**Figure 3A**). Tumor latency of WT mice fed the AIN93G and HFD was 17.4 weeks and 18.5 weeks, respectively (**Figure 3A**). The difference between *Mcp-1^-/-^* and WT mice was significant (*p* < 0.01), regardless of diet (**Figure 3A**).

Mice fed the HFD had greater tumor progression than mice fed the AIN93G diet (4679 ± 786% *vs.* 2430 ± 700%), regardless of genotype (**Figure 3B**). The tumor progression of *Mcp-1^-/-^* mice was lower than that of WT mice (2317 ± 901% *vs.* 4792 ± 545%), regardless of diet (**Figure 3B**). At the end of the study, mammary tumors from *Mcp-1^-/-^* mice weighed 64% less than that from WT mice (0.23 ± 0.15 g *vs.* 0.64 ± 0.09 g), regardless of diet (**Figure 3C**). There was no significant difference in tumor weight between the two diets, regardless of genotype (**Figure 3C**).

Few tumor-bearing mice in each group had detectable metastases in the lungs. The incidence of metastasis was 38% for *Mcp-1^-/-^* mice (5 out of 13 mice) and 39% for WT mice (14 out of 36 mice), regardless of diet. The incidence of metastasis was 50% for the HFD (13 out of 26 mice) and 26% for the AIN93G diet (6 out of 23 mice) regardless of genotype. There were no significant differences in these comparisons.

### Plasma Metabolomics Analysis

Plasma metabolomics analysis was performed to investigate effects of adipose MCP-1 deficiency and HFD on metabolome. Because few mice developed palpable mammary tumors, particularly *Mcp-1^-/-^* mice, the analysis was performed by using plasma samples from mice that did not develop palpable tumors. We identified 127 compounds from 467 discrete signals detected in plasma by the GC-TOF-MS. Eighty-seven of the 127 compounds met the criteria for statistical analysis (**Supplementary Table 1**). Fifty-six of these 87 compounds differed significantly among the four groups, including 22 differed by diet, 22 by genotype, and 34 by diet and genotype interactions (**Tables 2** **–** **4**).

**Table 2:** Identified plasma metabolites related to amino acid metabolism that differed in MMTV-PyMT mice with or without adipose monocyte chemotactic protein-1 knockout [*Mcp-1^-/-^* or wild-type (WT)] and fed the AIN93G or high-fat diet (HFD).

	AIN93G WT	AIN93G *Mcp-1^-/-^*	HFD WT	HFD *Mcp-1^-/-^*	Diet, *p*	Gene, *p*	Diet x Gene, *p*
Proteinogenic amino acids							
Alanine	1.00 ± 0.15^b^	1.74 ± 0.15^a^	1.24 ± 0.12^b^	1.19 ± 0.07^b^	0.21	0.01	<0.01
Glycine	1.00 ± 0.08	1.18 ± 0.07	0.97 ± 0.06	0.90 ± 0.08	0.04	0.45	0.09
Isoleucine	1.00 ± 0.07^b^	1.38 ± 0.09^a^	1.29 ± 0.06^a^	0.87 ± 0.04^b^	0.09	0.80	<0.01
Leucine	1.00 ± 0.07^b^	1.28 ± 0.08^a^	1.34 ± 0.08^a^	0.84 ± 0.05^b^	0.48	0.13	<0.01
Lysine	1.00 ± 0.12	1.40 ± 0.11	0.86 ± 0.06	1.25 ± 0.15	0.22	<0.01	0.99
Phenylalanine	1.00 ± 0.08^b^	1.37 ± 0.08^a^	1.25 ± 0.06^ab^	0.98 ± 0.09^b^	0.34	0.52	<0.01
Methionine	1.00 ± 0.06^b^	1.33 ± 0.08^a^	0.99 ± 0.07^b^	0.91 ± 0.10^b^	0.01	0.12	0.01
Proline	1.00 ± 0.20^b^	1.87 ± 0.27^a^	1.19 ± 0.20^ab^	0.92 ± 0.15^b^	0.08	0.16	0.01
Serine	1.00 ± 0.06	1.28 ± 0.08	0.96 ± 0.04	1.07 ± 0.10	0.09	0.01	0.24
Threonine	1.00 ± 0.09^b^	1.65 ± 0.16^a^	1.02 ± 0.07^b^	1.00 ± 0.08^b^	<0.01	<0.01	<0.01
Tyrosine	1.00 ± 0.06	1.30 ± 0.08	1.00 ± 0.04	1.35 ± 0.12	0.74	<0.01	0.77
Valine	1.00 ± 0.06^b^	1.43 ± 0.08^a^	1.28 ± 0.05^a^	0.92 ± 0.06^b^	0.09	0.59	<0.01
Nonproteinogenic amino acids and derivatives							
2-Aminobutyric acid	1.00 ± 0.24	1.61 ± 0.22	0.67 ± 0.06	0.69 ± 0.07	<0.01	0.07	0.08
Aminomalonate	1.00 ± 0.09	0.91 ± 0.11	0.66 ± 0.07	0.84 ± 0.10	0.04	0.66	0.15
2-Hydroxybutanoic acid	1.00 ± 0.17^a^	0.41 ± 0.05^b^	0.60 ± 0.15^ab^	0.70 ± 0.15^ab^	0.70	0.84	0.02
Indole-3-propionic acid	1.00 ± 0.37	1.81 ± 0.47	0.59 ± 0.15	0.52 ± 0.12	0.01	0.25	0.17
2-Ketoisocaproic acid	1.00 ± 0.14	1.13 ± 0.13	0.86 ± 0.06	0.65 ± 0.05	<0.01	0.71	0.11
Oxoproline	1.00 ± 0.07	1.17 ± 0.07	1.23 ± 0.06	1.01 ± 0.06	0.59	0.74	0.01
Taurine	1.00 ± 0.16	1.82 ± 0.32	1.78 ± 0.23	1.41 ± 0.19	0.44	0.33	0.01
Urea cycle metabolites							
Citrulline	1.00 ± 0.07	1.25 ± 0.09	0.89 ± 0.09	0.92 ± 0.06	0.01	0.08	0.17
Urea	1.00 ± 0.04^a^	0.42 ± 0.17^b^	0.47 ± 0.16^b^	0.79 ± 0.11^ab^	0.52	0.32	<0.01

Values of treatment groups are standardized to that of the AIN93G WT group. Values (means ± SEM) in the same row with different letters are significant at p ≤ 0.05 (false discovery rate-adjusted p values) (n = 10 per group).

**Table 3:** Identified plasma metabolites related to carbohydrate metabolism that differed in MMTV-PyMT mice with or without adipose monocyte chemotactic protein-1 knockout [*Mcp-1^-/-^* or wild-type (WT)] and fed the AIN93G or high-fat diet (HFD).

	AIN93G WT	AIN93G *Mcp-1^-/-^*	HFD WT	HFD *Mcp-1^-/-^*	Diet, *p*	Gene, *p*	Diet x Gene, *p*
1,5-Anhydroglucitol	1.00 ± 0.07^a^	0.79 ± 0.06^a^	0.34 ± 0.06^b^	0.40 ± 0.05^b^	<0.01	0.22	0.03
Erythritol	1.00 ± 0.09	1.32 ± 0.10	1.31 ± 0.11	1.09 ± 0.04	0.67	0.58	<0.01
Fumaric acid	1.00 ± 0.17^b^	2.48 ± 0.36^a^	1.35 ± 0.19^b^	1.11 ± 0.14^b^	0.03	0.01	<0.01
Glucose	1.00 ± 0.07^b^	0.98 ± 0.03^b^	1.05 ± 0.03^b^	1.24 ± 0.05^a^	<0.01	0.09	0.03
Glucose-1-phosphate	1.00 ± 0.14	0.79 ± 0.05	0.81 ± 0.07	1.23 ± 0.18	0.31	0.39	0.01
Glucuronic acid	1.00 ± 0.21^b^	3.59 ± 0.86^a^	1.13 ± 0.13^b^	1.09 ± 0.14^b^	0.01	0.01	0.01
Glycerol-α-phosphate	1.00 ± 0.11	1.09 ± 0.15	1.45 ± 0.12	1.06 ± 0.10	0.09	0.22	0.05
Glyceric acid	1.00 ± 0.18	1.74 ± 0.22	1.62 ± 0.23	1.28 ± 0.16	0.69	0.32	0.01
Hexuronic acid 1	1.00 ± 0.26^b^	3.63 ± 0.90^a^	1.11 ± 0.17^b^	1.04 ± 0.18^b^	0.02	0.01	0.01
Hexuronic acid 2	1.00 ± 0.10^b^	1.88 ± 0.33^a^	1.05 ± 0.04^b^	1.17 ± 0.08^ab^	0.07	0.01	0.04
3-Hydroxybutyric acid	1.00 ± 0.27	0.87 ± 0.13	1.03 ± 0.11	0.43 ± 0.06	0.22	0.03	0.16
Isocitric acid	1.00 ± 0.09^ab^	1.30 ± 0.13^a^	1.03 ± 0.12^ab^	0.83 ± 0.07^b^	0.04	0.62	0.03
Lactic acid	1.00 ± 0.28	1.65 ± 0.37	0.62 ± 0.06	0.57 ± 0.09	<0.01	0.21	0.16
Malic acid	1.00 ± 0.22^b^	2.52 ± 0.40^a^	1.07 ± 0.23^b^	1.00 ± 0.23^b^	0.01	0.01	0.01
Mannose	1.00 ± 0.07	1.08 ± 0.06	1.16 ± 0.07	0.94 ± 0.03	0.87	0.28	0.02
Myoinositol	1.00 ± 0.07^ab^	1.32 ± 0.09^a^	1.05 ± 0.11^ab^	0.95 ± 0.05^b^	0.07	0.21	0.02
Phosphate	1.00 ± 0.06^b^	1.07 ± 0.07^b^	1.49 ± 0.10^a^	0.95 ± 0.05^b^	0.02	<0.01	<0.01
Pyruvic acid	1.00 ± 0.20	1.42 ± 0.16	0.69 ± 0.10	0.88 ± 0.13	0.01	0.05	0.46
Sorbitol	1.00 ± 0.16	1.74 ± 0.21	1.07 ± 0.16	1.49 ± 0.09	0.57	<0.01	0.33
Succinic acid	1.00 ± 0.20	1.35 ± 0.21	1.54 ± 0.34	0.76 ± 0.14	0.91	0.37	0.02
Xylose	1.00 ± 0.04^b^	1.11 ± 0.07^ab^	1.26 ± 0.08^a^	1.03 ± 0.05^ab^	0.18	0.36	0.01

Values of treatment groups are standardized to that of the AIN93G WT group. Values (means ± SEM) in the same row with different letters are significant at p ≤ 0.05 (false discovery rate-adjusted p values) (n = 10 per group).

**Table 4:** Identified plasma metabolites related to lipid, nucleotide, and vitamin metabolisms that differed in MMTV-PyMT mice with or without adipose monocyte chemotactic protein-1 knockout [*Mcp-1^-/-^* or wild-type (WT)] and fed the AIN93G or high-fat diet (HFD).

	AIN93G WT	AIN93G *Mcp-1^-/-^*	HFD WT	HFD *Mcp-1^-/-^*	Diet, *p*	Gene, *p*	Diet x Gene, *p*
Lipid metabolism							
Cholesterol	1.00 ± 0.12	0.74 ± 0.08	1.32 ± 0.10	1.05 ± 0.08	<0.01	0.01	0.98
Diglycerol	1.00 ± 0.15	1.28 ± 0.11	1.42 ± 0.14	1.21 ± 0.08	0.16	0.80	0.05
Glycerol	1.00 ± 0.06	0.77 ± 0.05	1.05 ± 0.11	0.84 ± 0.03	0.38	<0.01	0.92
Heptadecanoic acid	1.00 ± 0.09	1.20 ± 0.04	1.27 ± 0.12	1.12 ± 0.05	0.24	0.79	0.04
Linoleic acid	1.00 ± 0.18	0.77 ± 0.09	1.24 ± 0.04	0.78 ± 0.08	0.29	<0.01	0.31
1-Monoolein	1.00 ± 0.13	0.76 ± 0.06	1.40 ± 0.18	0.99 ± 0.07	0.01	0.01	0.51
Myristic acid	1.00 ± 0.08	0.95 ± 0.07	0.88 ± 0.04	0.63 ± 0.08	<0.01	0.03	0.14
Oleic acid	1.00 ± 0.36^b^	2.37 ± 0.95^ab^	4.21 ± 0.67^a^	2.41 ± 0.64^ab^	0.02	0.75	0.02
Palmitoleic acid	1.00 ± 0.18	0.78 ± 0.10	0.30 ± 0.02	0.16 ± 0.02	<0.01	0.11	0.68
Nucleotides							
Pseudo uridine	1.00 ± 0.11	1.25 ± 0.08	1.33 ± 0.13	1.01 ± 0.07	0.66	0.72	0.01
Thymidine	1.00 ± 0.11	1.09 ± 0.10	0.67 ± 0.10	1.01 ± 0.11	0.06	0.05	0.25
Uric acid	1.00 ± 0.17	0.65 ± 0.12	1.06 ± 0.12	0.65 ± 0.08	0.82	0.01	0.82
Vitamins							
Threonic acid	1.00 ± 0.15	1.69 ± 0.22	1.76 ± 0.28	1.29 ± 0.12	0.37	0.59	<0.01
α-Tocopherol	1.00 ± 0.19	0.52 ± 0.11	1.50 ± 0.15	0.98 ± 0.15	<0.01	<0.01	0.91

Values of treatment groups are standardized to that of the AIN93G WT group. Values (means ± SEM) in the same row with different letters are significant at p ≤ 0.05 (false discovery rate-adjusted p values) (n = 10 per group).

**Figure 4:**
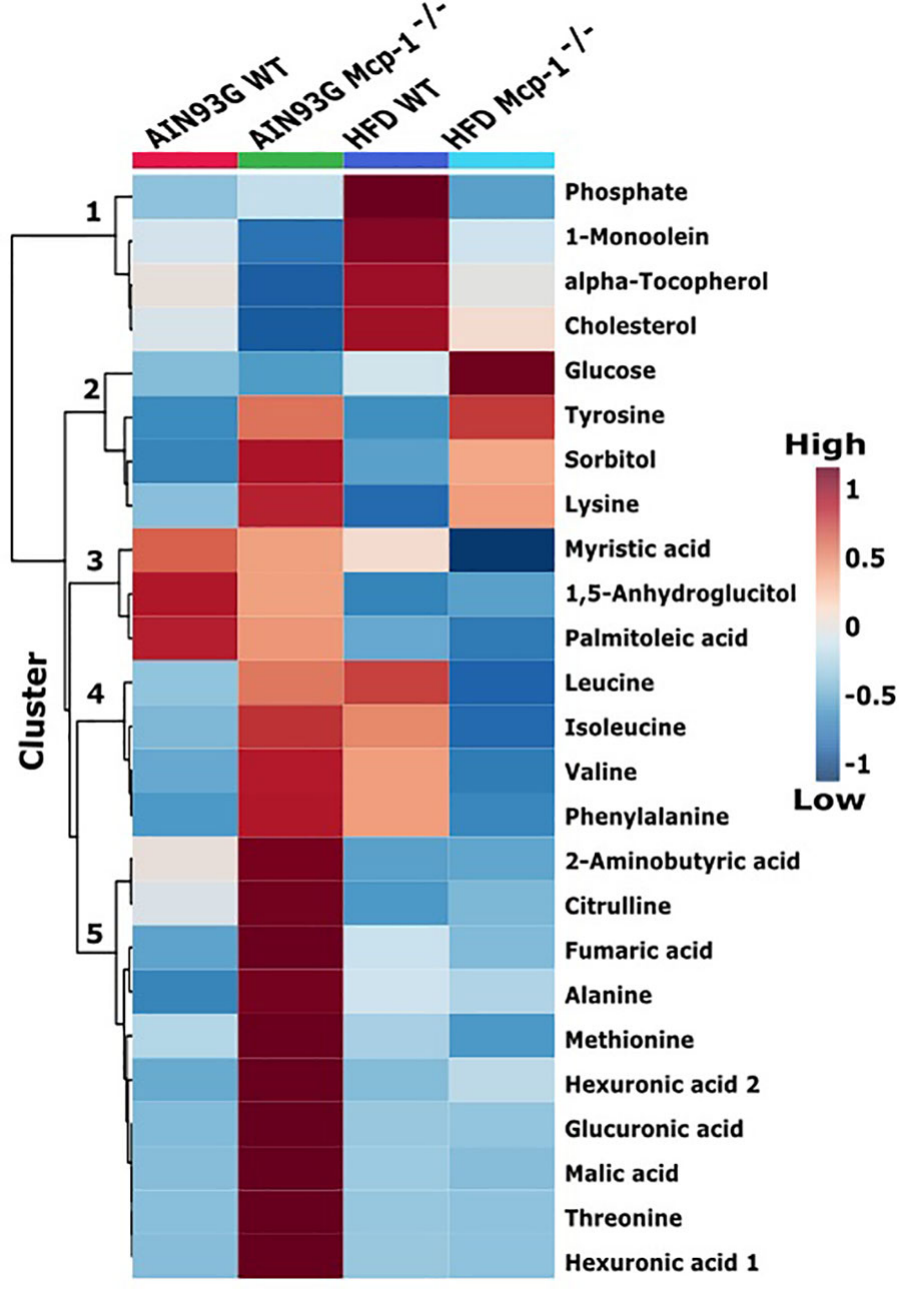
Hierarchical clustering heatmap of the top 25 plasma metabolites that differ among the four dietary groups. AIN WT (red): wild-type mice fed the AIN93G diet; AIN *Mcp-1^-/-^* (green): *Mcp-1^-/-^* mice fed the AIN93G diet; HFD WT (blue): wild-type mice fed the high-fat diet; HFD *Mcp-1^-/-^* (cyan): *Mcp-1^-/-^* mice fed the high-fat diet. Each cell on the map represents the group average of a metabolite (n = 10 per group).

The heatmap analysis of plasma metabolites provided intuitive visualization of the results. It produced five responsive clusters, 1) those that elevated in WT mice fed the HFD, 2) those that elevated in *Mcp-1^-/-^* mice regardless of diet, 3) those that remained relatively high in mice fed the AIN93G diet regardless of genotype, 4) those that elevated in *Mcp-1^-/-^* mice fed the AIN93G diet and WT mice fed the HFD, and 5) those that were higher in *Mcp-1^-/-^* mice fed the AIN93G diet compared to other groups (**Figure 4**).

**Figure 5:**
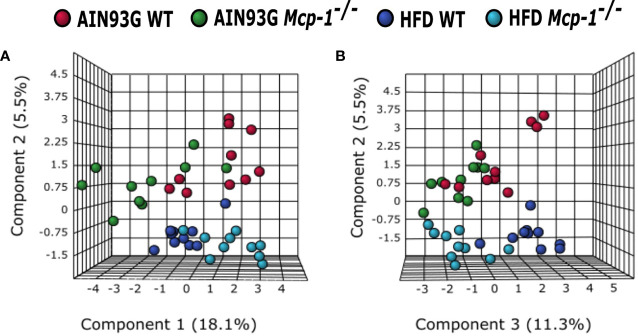
Sparse partial least square discriminant analysis of the plasma metabolome among the four dietary groups. **(A)** Components 1 *vs* 2; **(B)** Components 2 *vs* 3. AIN WT (red): wild-type mice fed the AIN93G diet; AIN *Mcp-1^-/-^* (green): *Mcp-1^-/-^* mice fed the AIN93G diet; HFD WT (blue): wild-type mice fed the high-fat diet; HFD *Mcp-1^-/-^* (cyan): *Mcp-1^-/-^* mice fed the high-fat diet (n = 10 per group).

**Figure 6:**
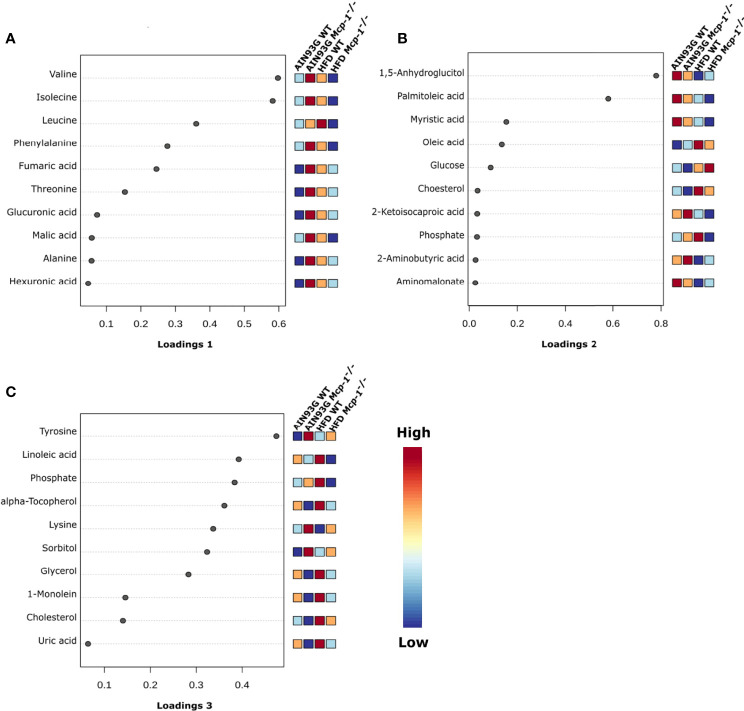
Loadings plots of the 10 metabolites that are the most influential in treatment separation among the four groups for components 1 **(A)**, 2 **(B)**, and 3 **(C)** (n = 10 per group).

The sPLSDA scores plot showed separations by diet and genotype (**Figures 5**, **6**). Along the x-axis, component 1 showed that WT mice fed the HFD and *Mcp-1^-/-^* mice fed the AIN93G diet were separated from WT mice fed the AIN93G diet whereas *Mcp-1^-/-^* mice fed the HFD remained similar to WT mice fed the AIN93G diet (**Figure 5A**). The loadings plot for component 1 identified the amino acids (alanine, isoleucine, leucine, phenylalanine, threonine, and valine) and carbohydrate metabolites (fumaric acid, glucuronic acid, hexuronic acid, and malic acid) as major determinants of separation (**Figure 6A**).

Along the y-axis, component 2 separated the groups into two categories based upon the diet, mice fed the AIN93G diet and mice fed the HFD, regardless of genotype (**Figure 5A**). The loadings plot for component 2 identified carbohydrate metabolites (1,5-anhydroglucitol, glucose, and phosphate), cholesterol, fatty acids (myristic acid, oleic acid, and palmitoleic acid), and amino acid derivatives (2-aminobutyric acid, aminomalonate, and 2-ketoisocapronic acid) as major determinants of separation (**Figure 6B**).

Along the x-axis, component 3 separated HFD-fed mice, but not AIN93G-fed mice, by genotype (**Figure 5B**). The loadings plot for component 3 identified amino acids (lysine and tyrosine), carbohydrate metabolites (phosphate and sorbitol), cholesterol, linoleic acid, lipid metabolites (glycerol and 1-monoolein), vitamin α-tocopherol and uric acid as major determinants of separation (**Figure 6C**).

### Pathway Analysis

**Figure 7:**
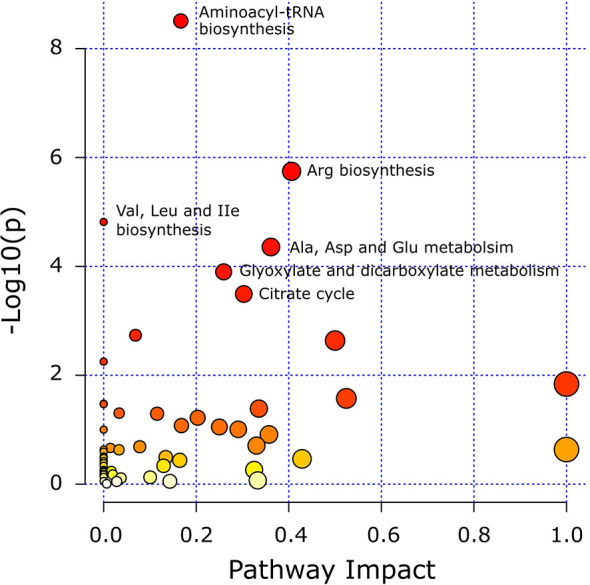
Matched metabolic pathway plot by using identified metabolites in MMTV-PyMT mice. The x-axis marks the pathway impact. The y-axis marks the pathway enrichment. Each node represents a pathway. The nodes with larger sizes and darker colors (from yellow to red) positioning towards top right region represent higher pathway impact values and higher pathway enrichment. Pathways that are significantly altered are presented with their names next to their nodes and are in **Table 5**. All detected metabolic pathways are presented in **Supplementary Table 2**.

Pathway analysis was conducted to determine metabolic pathways that were altered in MMTV-PyMT mice. The identified metabolites (**Supplementary Table 2**) were mapped into 55 metabolic pathways according to the KEGG database (35, 41). Six pathways were altered the most (**Table 5** and **Figure 7**). These included aminoacyl-tRNA biosynthesis, arginine biosynthesis, valine, leucine and isoleucine biosynthesis, alanine, aspartate and glutamate metabolism, glyoxylate and dicarboxylate metabolism, and citrate cycle (**Table 5**).

**Table 5:** Metabolic pathways identified by the pathway analysis that are significantly altered in MMTV-PyMT mice.

KEGG pathway	Number of metabolites identified	*p* ^a^	Impact^b^
Aminoacyl-tRNA biosynthesis	15	<0.01	0.17
Arginine biosynthesis	7	<0.01	0.41
Valine, leucine and isoleucine biosynthesis	5	<0.01	0
Alanine, aspartate and glutamate metabolism	8	<0.01	0.36
Glyoxylate and dicarboxylate metabolism	8	0.01	0.26
Citrate cycle	6	0.03	0.30

a p-values are obtained by the over-representation analysis and adjusted by the Holm method.

b Impact is the pathway impact score obtained by the pathway topology analysis.

### Network Analysis

**Figure 8:**
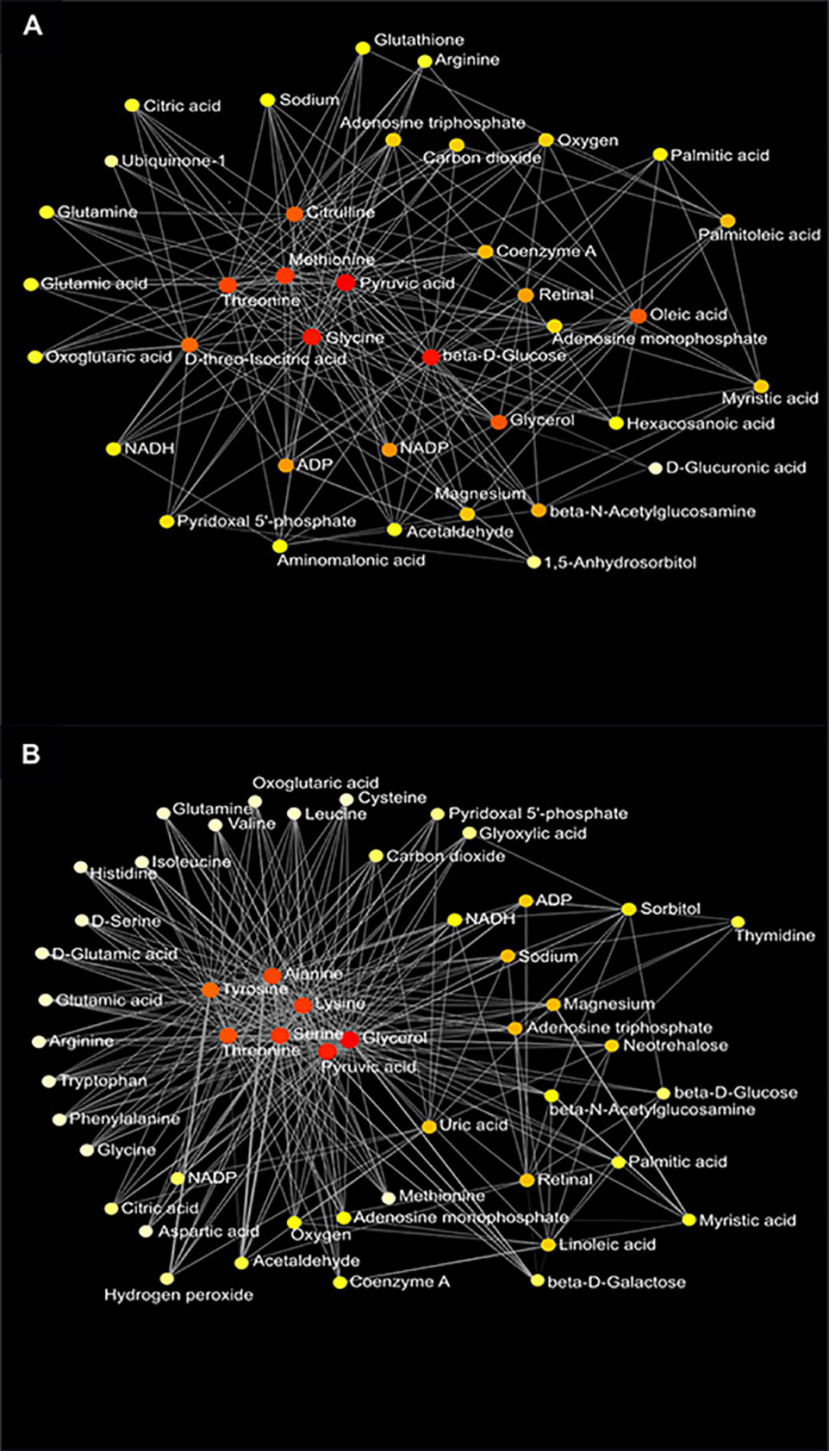
Metabolic network of plasma metabolites between the AIN93G and high-fat diet **(A)** and between wild-type and *Mcp-1^-/-^* mice **(B)**. Colors, from white-yellow to red, indicate levels of impact the metabolites have on the network in an ascending order (the number of connections a node has to other nodes and the number of shortest paths going through the node). Network statistics for **(A, B)** are presented in **Supplementary Table 3**.

**Figure 9:**
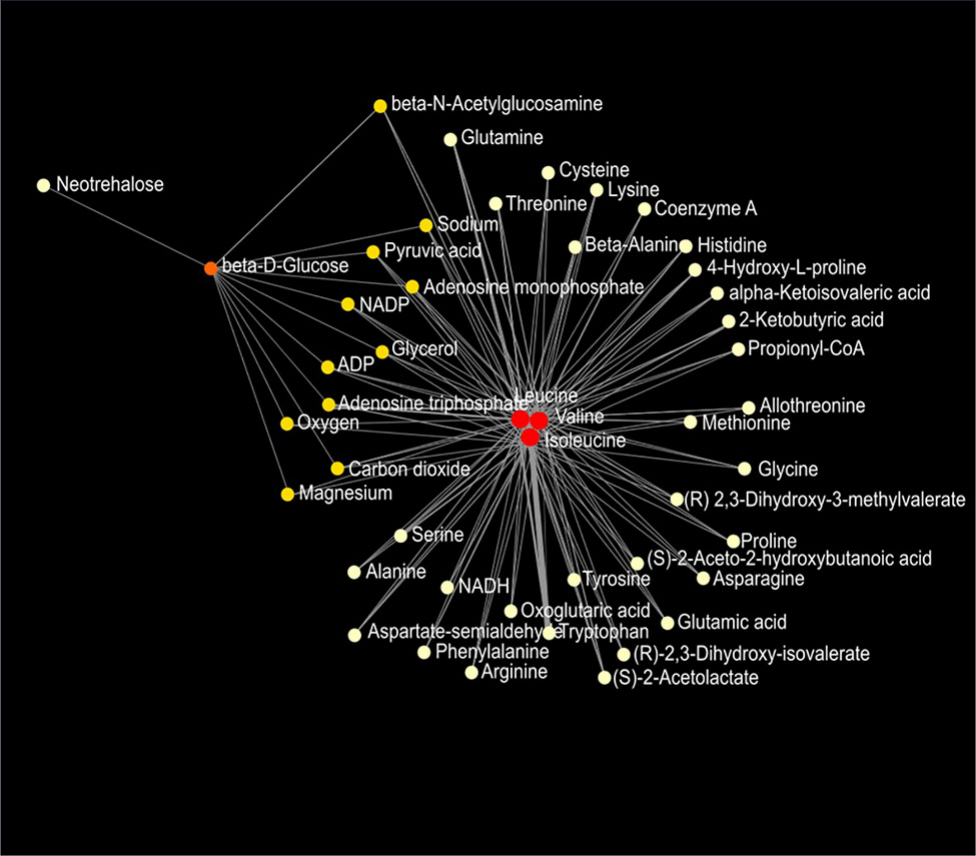
Metabolic network of plasma metabolites between wild-type and *Mcp-1^-/-^* mice fed the high-fat diet. Colors, from white-yellow to red, indicate levels of impact the metabolites have to the network in an ascending order (the number of connections a node has to other nodes and the number of shortest paths going through the node). Network statistics are presented in **Supplementary Table 3**.

Network analysis of plasma metabolites by using the KEGG Global Metabolic Network identified 9 metabolic pathways that differed between the AIN93G diet and HFD, regardless of genotype (**Table 6**). These pathways were glycine, serine, and threonine metabolism, valine, leucine, and isoleucine biosynthesis, arginine biosynthesis, citrate cycle, pyruvate metabolism, glycolysis/gluconeogenesis, alanine, aspartate, and glutamine metabolism, glyoxylate and dicarboxylate metabolism, and cysteine and methionine metabolism (**Table 6**). Nine pathways differed between WT and *Mcp-1^-/-^* mice, regardless of diet (**Table 6**). These included alanine, aspartate, and glutamine metabolism, glycine, serine, and threonine metabolism, tyrosine metabolism, citrate cycle, pyruvate metabolism, galactose metabolism, phenylalanine, tyrosine, and tryptophan biosynthesis, linoleic metabolism, and biotin metabolism (**Table 6**). Three pathways differed when HFD-fed *Mcp-1^-/-^* mice were compared to HFD-fed WT mice (**Table 6**). They were valine, leucine, and isoleucine biosynthesis, valine, leucine, and isoleucine degradation, and pantothenate and CoA biosynthesis (**Table 6**). Networks of the metabolites mapped by the Metabolite-Metabolite Interaction Network between AIN93G diet and HFD, between WT and *Mcp-1^-/-^* mice, and between the HFD-fed *Mcp-1^-/-^* and the HFD-fed WT mice are in **Figures 8A, B**, and **9**, respectively; their network statistics are in **Supplementary Table 3**.

**Table 6:** Metabolic pathways identified by the KEGG Global Metabolic Network that are significantly altered by the diet (AIN93G *vs.* high-fat diet) and genotype (wild-type *vs. Mcp-1^-/-^*) in MMTV-PyMT mice with or without adipose monocyte chemotactic protein-1 knockout (*Mcp-1^-/-^* or wild-type) and fed the AIN93G or high-fat diet.

Metabolic pathways	*p*
AIN93G diet *vs.* high-fat diet, regardless of genotype	
Glycine, serine, and threonine metabolism	<0.01
Valine, leucine, and isoleucine metabolism	<0.01
Arginine biosynthesis	<0.01
Citrate cycle	0.01
Pyruvate metabolism	0.01
Glycolysis/gluconeogenesis	0.02
Alanine, aspartate, and glutamate metabolism	0.03
Glyoxylate and dicarboxylate metabolism	0.03
Cysteine and methionine metabolism	0.03
Wild-type mice *vs. Mcp-1^-/-^* mice, regardless of diet	
Alanine, aspartate, and glutamine metabolism	<0.01
Glycine, serine, and threonine metabolism	<0.01
Tyrosine metabolism	0.02
Citrate cycle	0.02
Pyruvate metabolism	0.02
Galactose metabolism	0.05
Phenylalanine, tyrosine, and tryptophan biosynthesis	0.05
Linoleic acid metabolism	0.05
Biotin metabolism	0.05
Wild-type mice *vs. Mcp-1^-/-^* mice, high-fat diet	
Valine, leucine, and isoleucine biosynthesis	<0.01
Valine, leucine, and isoleucine degradation	<0.01
Pantothenate and CoA biosynthesis	0.05

## Discussion

The present study showed that adipose-derived MCP-1 contributed to mammary tumorigenesis in male MMTV-PyMT mice. We have three important findings with this male breast cancer model. First, mammary tumorigenesis is slow and less aggressive in male MMTV-PyMT mice. This is evidenced by a longer latency before detection of palpable mammary tumors (17.4 weeks) and a lower tumor incidence (56%) compared to female MMTV-PyMT mice reported to have a shorter latency (6 to 8 weeks) and 100% tumor incidence (24, 28, 43). The observed delay in onset of mammary tumorigenesis is consistent with a previous report showing a 14-week tumor latency with a 70% tumor incidence in male MMTV-PyMT mice (44). Findings from this study support clinical observations that breast cancer occurs late in life in men than that in women (9).

Second, mammary tumorigenesis in male MMTV-PyMT mice responds to dietary changes, evidenced by the increased tumor progression in mice fed the HFD. Mice fed the HFD gained body fat mass. Diet-induced obesity enhances mammary tumorigenesis in female MMTV-PyMT mice (24, 28, 43). Adipose tissue, considered an endocrine organ, produces proinflammatory cytokines that can be tumorigenic in rodent models of cancer, including the MMTV-PyMT model (24, 28, 43). Results from this study suggest that adipose-derived cytokines may be responsible, at least partly, for the HFD-enhanced mammary tumorigenesis in male mice.

Third, adipose-derived MCP-1 contributes to mammary tumorigenesis in male MMTV-PyMT mice. This is demonstrated by longer latency and lower tumor incidence, tumor progression, and tumor weight in *Mcp-1^-/-^* mice. These observations are MCP-1 specific and independent on body fat, because there were no differences in body fat mass between *Mcp-1^-/-^* and WT mice, particularly those fed the HFD. These findings are consistent with a previous report that adipose MCP-1 deficiency mitigates mammary tumorigenesis in female MMTV-PyMT mice (24).

In this study, we analyzed plasma metabolome in male MMTV-PyMT mice. Pathway analysis showed disturbances in metabolic pathways in MMTV-PyMT mice. The two most significant alterations are pathways related to amino acid and carbohydrate metabolisms. The aminoacyl-tRNA biosynthesis pathway is important in protein synthesis (45). It is a group of aminoacyl-tRNA synthetases that catalyze aminoacylations by covalently linking an amino acid to its cognate tRNA in the first step of protein translation. This includes glycine, serine, and threonine metabolism, valine, leucine and isoleucine biosynthesis, arginine biosynthesis, alanine, aspartate and glutamate metabolism, and phenylalanine, tyrosine, and tryptophan biosynthesis identified by the network analysis in MMTV-PyMT mice.

Alteration in the citrate cycle and pyruvate metabolism is an evidence of disturbed carbohydrate metabolism. However, it is interest to find the glyoxylate and dicarboxylate metabolism pathway in MMTV-PyMT mice. The glyoxylate cycle, a modification of the citrate cycle, was found in plants and some microorganisms but not in animals because animals lack two key enzymes of the cycle (isocitrate lyase and malate synthase). However, available studies have showed that the glyoxylate cycle occurs in animals (46, 47). The detection of isocitric acid and malic acid in plasma of MMTV-PyMT mice support the existence of the glyoxylate cycle in these mice. The potential involvement of glyoxylate and dicarboxylate metabolism in altered carbohydrate metabolism in MMTV-PyMT mice and its impact on mammary tumorigenesis certainly warrant further investigation.

Results from the network analysis support findings from the pathway analysis. It further demonstrates that both the HFD and adipose MCP-1 deficiency alter amino acid and carbohydrate metabolisms in male MMTV-PyMT mice. It is particularly interesting that both branched-chain amino acid (BCAA) biosynthesis and degradation pathways were altered when HFD-fed *Mcp-1^-/-^* mice were compared to their WT counterparts. Our findings of accelerated BCAA metabolism support the clinical observations of disturbed BCAA metabolism in human breast cancer patients (48, 49).

Heatmap analysis illustrates the top 25 metabolites that are major determinants in separation of the four dietary groups. The expression of 17 of them was higher in AIN93G-fed *Mcp-1^-/-^* mice than in their WT counterparts. These include essential amino acids (isoleucine, leucine, lysine, methionine, phenylalanine, threonine, and valine), nonessential amino acids (alanine and tyrosine), amino acid metabolites (2-aminobutyric acid and citrulline), metabolites of carbohydrate metabolism (fumaric acid, glucuronic acid, malic acid, and sorbitol), and fatty acid metabolite hexuronic acids. These findings indicate that MCP-1 deficiency may have altered metabolism in AIN93G-fed mice, particularly amino acid metabolism, and that adipose-derived MCP-1 may contribute to metabolic homeostasis in mice fed a non-obesogenic diet. It is interest to note that the expression of 14 of these 17 metabolites (except lysine, sorbitol, and tyrosine) were lower in HFD-fed *Mcp-1^-/-^* mice. It suggests that a high-energy intake may attenuate, at least partly, the metabolic alterations caused by adipose MCP-1 deficiency.

Multivariate and clustered heatmap analyses showed the separation of plasma metabolome among the four groups. Component 1 of the sPLSDA scores plot showed that clusters of WT mice differed by diet. However, the cluster of HFD-fed *Mcp-1^-/-^* mice remained similar to that of AIN93G-fed WT controls. This is the case for the major determinant metabolites shown in the loadings plot for component 1, in which BCAAs and phenylalanine are major determinants that separate the dietary groups. BCAAs account for one-third dietary essential amino acids and make up 20% of total protein content (50). BCAAs, specifically leucine, activate mammalian target of rapamycin complex (mTORC) signal pathway that is essential for initiation of protein synthesis (51, 52). Elevated expression of BCAAs in HFD-fed WT mice, compared to that in AIN93G-fed WT mice, are consistent with findings from both human and rodent studies (53, 54) and support the concept that the impaired BCAA catabolic pathway in obesity leads to BCAA buildup in the blood (53, 54). Significantly lower expression of BCAAs in HFD-fed *Mcp-1^-/-^* mice, compared to that in HFD-fed WT mice, indicates that adipose MCP-1 deficiency may attenuate the impaired BCAA metabolism in mice fed an obesogenic diet.

The elevated expression of four metabolites (cholesterol, phosphate, 1-monoolein, and α-tocopherol) identified by the heatmap in HFD-fed WT mice suggests accelerated metabolism in mice fed an obesogenic diet. Phosphate is involved in many anabolic and catabolic metabolisms. Cholesterol is essential for membrane biogenesis and a precursor for steroid hormone synthesis. Cholesterol is elevated in mammary tumors in MMTV-PyMT mice fed an HFD (55). Hypercholesterolemia promotes mammary tumorigenesis in MMTV-PyMT mice (56, 57). Elevations in α-tocopherol (an essential nutrient) and 1-monoolein (a major hydrolysis product of dietary triacylglycerol) (58) indicate an increase in nutrient uptake from the diet and an up-regulation in lipolysis of triacylglycerol in these mice. The attenuated expression of these metabolites in HFD-fed *Mcp-1^-/-^* mice suggests that adipose-derived MCP-1 may contribute to the diet-induced metabolic dysregulation in WT mice.

The significant elevation in glucose in HFD-fed *Mcp-1^-/^* ^-^ mice suggests impaired glucose metabolism under the MCP-1 deficiency in mice consuming an obesogenic diet. This elevation is not solely due to MCP-1 deficiency nor HFD alone, because such elevation was not observed in *Mcp-1^-/-^* mice fed the AIN93G diet nor WT mice fed the HFD. Rather, it is likely an interaction between MCP-1 deficiency and the HFD. The impairment is supported by the lower expression of 1,5-anhydroglucitol in these HFD-fed *Mcp-1^-/-^* mice. 1,5-Anhydroglucitol is a marker of glycemic control; its blood level is inversely correlated with blood glucose (59). Cancer cells demand high glucose uptake for their rapid, uncontrolled proliferation (60). The roles of adipose-derived MCP-1 in glucose metabolism under the obesogenic condition and its impact on mammary tumorigenesis warrant further investigation.

We found there were no differences in plasma concentrations of MCP-1 between *Mcp-1^-/-^* and WT mice fed the AIN93G diet. In this study, the expression of *Mcp-1* in adipose tissue was low in *Mcp-1^-/-^* mice. However, it does not exclude the possibility that MCP-1 from non-adipose tissue may contribute to plasma concentrations in mice consuming the AIN93G diet. Nevertheless, significant decreases in plasma MCP-1 in HFD-fed *Mcp-1^-/-^* mice, compared to their WT counterparts, demonstrates the validity of the model and that decreases in plasma MCP-1 are a result of MCP-1 deficiency in adipose tissue.

A limitation of this study is that we were not able to analyze plasma metabolome from tumor-bearing mice because few mice, particularly *Mcp-1^-/-^* mice, developed palpable mammary tumors. This made us unable to examine the metabolic profile in the presence of mammary tumor. Furthermore, cautions should be taken in data interpretation, as the observed plasma alterations could be results from undetected nonpalpable tumors, stromal or systematic changes, or their combinations. Nevertheless, this study showed plasma metabolome in mice carrying PyMT oncogene and its changes resulted from adipose *Mcp-1* knockout and high-fat consumption. To our knowledge, this is the first study providing an assessment of plasma metabolic profile in this male MMTV-PyMT breast cancer model. Metabolomics differences between mammary tumors and mammary glands and the resulting systematic changes by adipose MCP-1 deficiency certainly warrant further investigation.

In summary, the present study showed that adipose-specific MCP-1 deficient mice had longer tumor latency and lower tumor incidence, tumor progression, and tumor burden compared to WT mice. It indicates that adipose-derived MCP-1 may contribute to mammary tumorigenesis in male MMTV-PyMT mice. Plasma metabolomics analysis identified 56 metabolites that differed significantly among the four dietary groups. Pathway and network analyses of the identified metabolites showed that amino acid and carbohydrate metabolisms are the most disturbed pathways in MMTV-PyMT mice. These metabolomics findings warrant further investigation on the role of adipose-derived MCP-1 in causal relationships between cancer metabolism and mammary tumorigenesis with this MMTV-PyMT model and through which to build strategies for prevention and treatment of male breast cancer.

## Data Availability Statement

The original results for this study are presented in the article/**Supplementary Material**. Further inquiries can be directed to the corresponding author.

## Ethics Statement

The animal study was reviewed and approved by The Institutional Animal Care and Use Committee, Grand Forks Human Nutrition Research Center, Grand Forks, ND, USA.

## Author Contributions

LY and SS designed the study, conducted experiments, and collected and analyzed data. All authors contributed to data interpretation, wrote and revised the manuscript, and agreed to be accountable for the content of the work.

## Funding

This work was funded by the USDA Agricultural Research Service Projects #3062-51000-050-00D and #3062-51000-056-00D.

## Conflict of Interest

The authors declare that the research was conducted in the absence of any commercial or financial relationships that could be construed as a potential conflict of interest.

## Publisher’s Note

All claims expressed in this article are solely those of the authors and do not necessarily represent those of their affiliated organizations, or those of the publisher, the editors and the reviewers. Any product that may be evaluated in this article, or claim that may be made by its manufacturer, is not guaranteed or endorsed by the publisher.
